# Evaluation of Portable Point-of-Care CD4 Counter with High Sensitivity for Detecting Patients Eligible for Antiretroviral Therapy

**DOI:** 10.1371/journal.pone.0034319

**Published:** 2012-04-19

**Authors:** Yukari C. Manabe, Yaping Wang, Ali Elbireer, Brandon Auerbach, Barbara Castelnuovo

**Affiliations:** 1 Infectious Diseases Institute, Makerere College of Health Sciences, Kampala, Uganda; 2 Division of Infectious Diseases, Department of Medicine, Johns Hopkins University School of Medicine, Baltimore, Maryland, United States of America; 3 Makerere University-Johns Hopkins University Clinical Core Laboratory, Department of Pathology, Johns Hopkins University School of Medicine, Baltimore, Maryland, United States of America; 4 Division of Pathology, Department of Medicine, Johns Hopkins University School of Medicine, Baltimore, Maryland, United States of America; Boston University, United States of America

## Abstract

**Background:**

Accurate, inexpensive point-of-care CD4+ T cell testing technologies are needed that can deliver CD4+ T cell results at lower level health centers or community outreach voluntary counseling and testing. We sought to evaluate a point-of-care CD4+ T cell counter, the Pima CD4 Test System, a portable, battery-operated bench-top instrument that is designed to use finger stick blood samples suitable for field use in conjunction with rapid HIV testing.

**Methods:**

Duplicate measurements were performed on both capillary and venous samples using Pima CD4 analyzers, compared to the BD FACSCalibur (reference method). The mean bias was estimated by paired Student's t-test. Bland Altman plots were used to assess agreement.

**Results:**

206 participants were enrolled with a median CD4 count of 396 (range; 18–1500). The finger stick PIMA had a mean bias of −66.3 cells/µL (95%CI −83.4−49.2, *P*<0.001) compared to the FACSCalibur; the bias was smaller at lower CD4 counts (0–250 cells/µL) with a mean bias of −10.8 (95%CI −27.3−+5.6, *P = *0.198), and much greater at higher CD4 cell counts (>500 cells/µL) with a mean bias of −120.6 (95%CI −162.8, −78.4, *P*<0.001). The sensitivity (95%CI) of the Pima CD4 analyzer was 96.3% (79.1–99.8%) for a <250 cells/ul cut-off with a negative predictive value of 99.2% (95.1–99.9%).

**Conclusions:**

The Pima CD4 finger stick test is an easy-to-use, portable, relatively fast device to test CD4+ T cell counts in the field. Issues of negatively-biased CD4 cell counts especially at higher absolute numbers will limit its utility for longitudinal immunologic response to ART. The high sensitivity and negative predictive value of the test makes it an attractive option for field use to identify patients eligible for ART, thus potentially reducing delays in linkage to care and ART initiation.

## Introduction

The availability of highly active antiretroviral therapy (ART) to the developing world has been life-saving and led to remarkable reversals in mortality rates and opportunistic infection incidence rates. [1],[2] According to the WHO classification adopted by most countries in Sub-Saharan Africa, clinicians base the decision to initiate antiretroviral therapy (ART) on CD4+ cell count or WHO stage IV status criteria. [3] Initiating ART in asymptomatic patients with higher CD4+ T cell counts who may not qualify by clinical criteria is also desirable as this avoids both morbidity and mortality. [4], [5] Eligibility for ART is very difficult to assess by clinical criteria only; many patients eligible by CD4 T cell criteria may not receive medication if clinical signs and symptoms only are used. [6], [7].

CD4+ T cell counts often need to be done at higher-level health centers with functional labs and adequate power source, or at reference regional labs. Because the number of facilities that offer a CD4+ T cell count is limited, there are often delays in obtaining the test results and, subsequently, delays in the initiation of ART. Furthermore, in most ART rollout programs in sub-Saharan Africa (SSA), viral load testing is not available, and immunologic monitoring is used instead as part of routine follow-up care.

Even though financial support continues to be a principal factor in addressing HIV/AIDS related mortality in developing countries, a more fundamental implementation obstacle is the inadequate access to basic health services. [8] Due to limited laboratory capacity, access to diagnostic testing is particularly inadequate, and is often non-existent in rural settings. [9] Point-of-care (POC) instruments could provide diagnostic capacity in resource-limited settings. Accurate, inexpensive point-of-care CD4+ T cell testing technologies are urgently needed. Recently, several point-of-care CD4+ T cell platforms that can deliver CD4+ T cell results at lower level health centers or even at community outreach with voluntary counseling and testing have emerged. Many of these new POC instruments are battery operated, use venous and/or finger-stick blood samples and could positively impact delays in ART initiation. The translational research team at the Infectious Diseases Institute (IDI) in Kampala Uganda, in collaboration with the MU-JHU Core Lab at the IDI, evaluated a point-of-care CD4+ T cell counter, the Pima CD4 Test System, a portable bench-top instrument that is designed to perform in laboratory as well in non-laboratory environments.

## Methods

### Ethics Statement

The protocol was approved by the Scientific Review Committee of the Infectious Diseases Institute, Joint Clinical Research Center Institutional Review Board, and the Uganda National Council of Science and Technology.

### Population tested and study design

Patients at the Adult Infectious Diseases Institute (IDI) Clinic within the Mulago Hospital Complex in Kampala, Uganda were approached during a routine clinic visit. Patients were invited to take part in the study and gave written informed consent before being enrolled. Measurements were performed using a total of 4 Pima CD4 analyzers (Alere Inc.Waltham, Maryland, USA). Duplicate measurements were performed on both capillary and venous samples using two different Pima CD4 analyzers. Capillary samples were obtained by a study nurse; the participant's finger was lanced and blood was collected from the finger tip directly into the cartridge. Venous samples were collected by venipuncture and stored in an EDTA tube at room temperature. At the time of analysis (within 6 hours of collection), the tube was inverted 10–15 times before each measurement to ensure proper sample mixing, and then 10 μl of mixed blood was pipetted into the capillary tube at the end of the cartridge.

Duplicate measurements on a single venous blood sample were also performed using the BD FACSCalibur (Becton Dickinson, CA, USA) as a reference method. Testing on both BD FACSCalibur instruments was performed within 24 hours of blood collection. Since the Pima CD4 Test System was strictly for investigational use, patients' clinicians were provided only with the CD4+ T cell results from the FACSCalibur reference method, while the results of the PIMA analyzers were stored in the study files.

### Pima CD4 system and CD4 enumeration

The self-contained disposable Pima CD4 test cartridge uses an integrated capillary to capture 5 µL of sample and contains all dried reagents needed to perform the test. The test is performed entirely within the cartridge and no part of the Pima Analyzer has contact with the sample at any time, thus minimizing the risk of analyzer contamination and sample carry-over. After inserting the cartridge into the analyzer, the sample is transported by peristaltic movement into an incubation compartment and allowed to interact with CD3 and CD4 surface antigens (both carried by T helper lymphocytes) specific antibodies labeled with PE and PE-Cy5, respectively. After an automated defined incubation time, the stained sample is transferred into a reading compartment of the cartridge. Fluorescence signals from the bound antibodies are then detected by a CCD camera and analyzed by an on-board, embedded computer. Results were displayed by the instrument as the absolute number of cells/µL. Results are also stored and can be printed any time after the test.

The reference method was the BD FACSCalibur using the MultiTEST CD3/CD8/CD45/CD4 reagent with TruCOUNT Tubes, and MultiSET software. These tests were done by Makerere University-Johns Hopkins University Clinical Core laboratory, a College of American Pathologists (CAP) certified laboratory that undergoes external proficiency testing through the College of American Pathologists and UKNEQAS as well as daily calibration and internal quality controls.

### Statistical Analysis Methods

Data were analyzed using SAS 9.2 software (SAS Institute Inc. North Carolina, USA). The Pearson Correlation Coefficient was calculated for each pair of Pima capillary tests or Pima venous tests and the BD FACSCalibur gold standard tests; the relative difference analysis was performed for each pair of tests by Wilcoxon signed-rank test since we could not assume that the population was normally distributed, and the mean bias was estimated by paired Student's t-test. Using the first CD4 count measurement of each pair, scatter plots and Bland-Altman plots [10] were used to assess the agreement between the Pima capillary and the BD FACSCalibur results, and between the Pima venous and the BD FACSCalibur results.

## Results

### Characteristics of the study population

Two hundred and six unselected participants were enrolled in the study between September 7 and November 2, 2009. The majority were women (156, 75.7%), the median age was 36 years (range 18–68), and 49% of the patients were WHO stage 3 or 4. The median absolute CD4+ T cell count by FACSCalibur was 396 (range: 18–1500). 142 (68.9%) were currently on ART.

### Performance of Pima CD4 analyzer

Duplicate measurements were performed on venous blood samples using two different Pima CD4 analyzers on 206 participants. A total of 446 tests were performed; of these, 36 tests (8.1%) generated an error instead of a valid result. Samples yielding an error instead of a valid result were retested. Four hundred-ten results (91.93%) were used for statistical analysis. Duplicate measurements were performed on finger stick samples again using two different Pima analyzers on only 176 patients due to errors or refusal to have multiple finger sticks. On capillary blood, a total of 372 tests were performed; of these, 66 tests (17.74%) generated an error instead of a valid result. Three hundred-six results (82.26%) were used for statistical analysis.

### Comparison of the Pima capillary and venous testing methods to BD FACSCalibur

**Figure 1:**
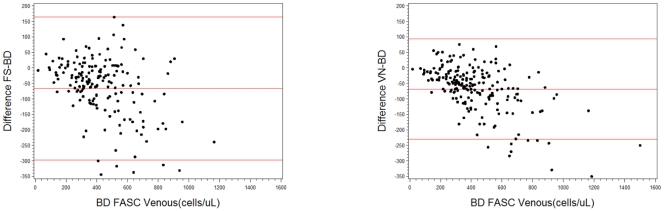
Bland Altman analyses of Pima test results compared to BD FASCalibur method. a) Pima capillary samples and, b) Pima venous samples, versus the BD FACS reference method. Upper and lower lines show 2 standard deviations from the mean (center line).

Paired capillary PIMA and FACSCalibur results were compared using Bland-Altman analysis. The plots are shown in Figure 1. The mean bias of the capillary testing was −66.3 cells/µL (95%CI −83.4−49.2, *P*<0.001) overall for the 176 paired samples; the bias was smaller at lower CD4 counts (0–250 cells/µL) with a mean bias of −10.8 (95%CI −27.3−+5.6, *P = *0.198), and much greater at higher CD4 cell counts (>500 cells/µL) with a mean bias of −120.6 (95%CI −162.8, −78.4, *P*<0.001). (Table 1) The correlation coefficient between 176 capillary and BD FACSCalibur paired tests was 0.86 (*P*<0.001).

**Table 1:** Mean and Median Bias Analysis of Pima Capillary and Venous Measurements.

Comparison	Reference method* (cells/µl)	Number of pairs (%)	Mean Bias (95%CI)	Median Bias (IQR)	*P-*value**
Pima CD4 Capillary	Overall	176	−66.3(−83.4, −49.2)	−41.0(−105.0, 4.5)	<0.001
	0–250	27	−10.8(−27.3, 5.6)	−6.0 (−32.0, 11.0)	0.198
	250–500	90	−47.3(−62.2, −32.5)	−40.5 (−76, −5.0)	<0.001
	>500	59	−120.6 (−162.8, −78.4)	−107.0 (−197.0, −10.0)	<0.001
Pima CD4 Venous	Overall	206	−68.5 (−79.6, −57.4)	−54.0 (−95.0, −17.0)	<0.001
	0–250	35	13.6 (2.5, 24.7)	−16.0 (−35.0, −1.0)	0.020
	250–500	107	−54.6 (−64.5, −44.7)	−53.0 (−79.0, −23.0)	<0.001
	>500	64	−121.7 (−147.9, −95.4)	−93.5 (−167.5, −64.5)	<0.001

* BD FACSCalibur.

** *P-*value using Wilcoxon Signed Rank Test.

CI = confidence interval, CD4 = CD4+ T cell count (cells/µL).

**Figure 2:**
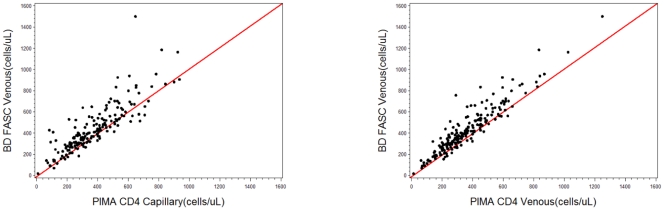
Comparison of Pima CD analyzer capillary samples testing (a) and venous samples testing (b) compared to BD FACS reference method.

The comparison of the Pima venous samples compared to the BC FACSCalibur showed similar results. (Table 1) The mean bias of the venous testing was −68.5 cells/µl (95%CI −79.6−57.4, *P*<0.001) overall for the 176 paired samples, and smaller at lower CD4 counts (0–250 cells/µL) with a mean bias of 13.6 cells/µl (95%CI 2.52−24.7, *P = *0.02) and much greater at higher CD4 T cell counts (>500 cells/µL) with a mean bias of −121.7 (95%CI −147.9 – −95.4, *P*<0.001). The correlation coefficient between 206 venous and BD FACSCalibur paired tests was 0.93 (*P*<0.001). (See Figure 2) Although a simple linear regression could be fitted with the data, whereby the BD result could be predicted on the basis of the Pima result, the negative predictive value of the predicted result decreased compared to the measured BD value and would not reliably improve the predictive accuracy of the test.

The Pima venous compared to the Pima capillary testing showed that the values were similar overall with a mean bias of −3.8 cells/µl (95%CI −17.75, 10.25, *P* = 0.598). The correlation between the two tests was 0.87 but was not statistically significant (*P* = 0.39).

### Agreement and precision analysis based on duplicate measurements

Duplicate testing was only possible for 130 patients with Pima capillary due to the high error rate and the interclass correlation coefficient between the 2 readings was 0.79. For the venous samples, there were 204 patients who had 2 duplicate measurements, and all 206 patients had duplicate FACSCalibur samples (both rho = 0.96). Table 2 shows the precision of the duplicate testing and shows a significant difference in the Pima capillary duplicates (mean difference 33.8±9.8 cells/µL, *P = *0.005), but acceptable precision for both the Pima venous and BD FACSCalibur gold standard.

**Table 2:** Precision of duplicate testing for all 3 methods.

Test Type	Number of pairs	MeanDifference	95% CI	P-value*
Pima Capillary	130	33.38	14.19, 52.57	0.005
Venous	204	−6.54	−13.69, 0.61	0.099
BD FACS Venous	206	1.87	−6.77, 10.51	0.899

Using Wilcoxon Signed Rank Test;

CI  = Confidence Interval.

### Sensitivity, specificity of venous and capillary samples in assessing eligibility for ART

We assessed the sensitivity and specificity of the Pima CD4 analyzer using a cut-off of 250 cells/µL and 300 cells/µL. As shown in Table 3 the sensitivity of the Pima CD4 analyzer was high for both cut-offs with very high negative predictive values, making the Pima device an useful tool to identify patients in need of ART, although some ineligible patients may be referred for ART.

**Table 3:** Sensitivity, specificity, negative predictive value, and positive predictive value of the Pima CD4 test (finger stick and venous) compared to the reference method BD FACSCalibur.

Pima CD4 Test	Sensitivity (95%CI)	Specificity (95%CI)	NPV (95%CI)	PPV (95%CI)
Finger stick <250 cells/µl	0.963 (0.791−0.998)	0.866 (0.798−0.914)	0.992 (0.951−0.999)	0.565 (0.412−0.708)
Finger stick <300 cells/µl	0.932 (0.803−0.982)	0.795 (0.715−0.859)	0.972 (0.915−0.993)	0.603 (0.477−0.717)
Venous <250 cells/µl	0.943 (0.795−0.990)	0.854 (0.789−0.901)	0.986 (0.947−0.998)	0.569 (0.433−0.696)
Venous <300 cells/µl	0.982 (0.892−0.999)	0.753 (0.675−0.818)	0.991 (0.945−0.999)	0.598 (0.490−0.697)

CI = confidence interval, NPV = negative predictive value, PPV = positive predictive value.

## Discussion

This evaluation of the Pima CD4 point-of-care test shows that there was significant bias toward lower absolute CD4+ T cell counts for both venous and capillary finger stick Pima methods in comparison to the BD FACSCalibur measurement from a CAP certified lab. The bias was lower at lower absolute CD4+ T cell counts. This would limit the usefulness of the Pima CD4 analyzer for longitudinal care and immunological monitoring. Our data corroborates that of three other studies where the Pima CD analyzer was less accurate at higher CD4 cell counts, was consistently negatively biased, although to different degrees compared to our study. [11], [12], [13] In the study from Senegal which also included HIV-negative patients allowing for higher CD4 ranges, this increasing negative bias and decrease in the % similarity with FACSCount results with increasing CD4 T cell counts was particularly evident. The other published study of the Pima analyzer showed little bias at both high and low CD4+ T cell counts. [14].

The machine is highly portable, self-contained and battery-operated and easy to use by most health care worker cadres. [14] The staff in our study were trained in a single 2 hour session. Quality control and observed practical training would be required to ensure that good volume and flow of blood is obtained. Without this, the accuracy of the test is compromised. As a screening test for ART eligibility in the field, however, both the venous and Pima capillary finger stick methods showed good negative predictive value. This test would identify almost all eligible patients to be referred at the time of HIV testing, thereby eliminating the delay between a positive HIV test and CD4+ T cell count testing and result reporting back to the patient. The literature on outcomes of HIV/AIDS care programs is often restricted to patients on antiretroviral therapy. However, patients are often diagnosed with HIV infection at community-based counseling and testing sites, and then required to travel to health centers offering HIV care on at least 2 occasions to get a CD4+ T cell count and receive the results. Both of these steps have significant potential for delays, loss to follow-up and death.

Cohorts from South Africa Mozambique, Kenya, and Uganda have reported on outcomes and waiting times during the interval between enrollment in an ART program and treatment initiation. [15], [16], [17], [18], [19], [20], [21] In particular, 2 cohorts from South Africa and Mozambique demonstrated high rates of lost-to-follow-up prior to ART initiation. In Durban, South Africa, 45% of persons registering at an ART clinic with a newly diagnosed HIV infection were lost to care. [21] In another study of 7,005 newly diagnosed HIV patients in Mozambique, only 57% registered at an ART clinic within one month, and 77% of ART clinic registrants received a CD4+ T cell count test within one month of registration. Only 49% of those patients who received a CD4+ T cell count initiated treatment; there was a median of 71 days between receiving their results and starting ART. [19] The Pima test was recently examined in 929 patients from 4 clinics in Mozambique randomized to either lab-based CD4 testing (492 participants) or point-of-care Pima CD4 testing (437 participants). There was a significant decrease in losses to follow up between enrolment and antiretroviral therapy initiation from 64% to 33% (adjusted odds ratio [OR] 0.27, 95% confidence intervals [CI] 0.21–0.26). [22]

The finger stick platform is very attractive, but at the time of this testing still problematic; the number of patients who had errors with finger stick testing with particular cartridge lots was rate-limiting. This has also been noted in the studies from both Thailand and Senegal. [12], [13] Since then, the company has made some modifications of the cartridge including increasing the concentration of EDTA. If these high error rates are not reduced in subsequent cartridge models, technician time required to run a single test would increase as well as the per test cost. In addition, technicians need to be trained as differences in methods of elucidating finger stick blood can lead to differences in the measured values of CD4 because the test measures absolute values rather than percentages. Although we consistently saw negative bias accentuated at higher CD4 T cell counts, a study in South Africa reported negative bias with decreased precision (greater variation) with capillary sampling which was operator-dependent and particularly pronounced in clinics with less laboratory capacity. [23] The quality of the capillary blood flow was a notable issue; in order to establish an adequate amount of blood at a fast enough rate to fill the capillary tube on the cartridge, a larger lancet is recommended. Another limitation of the device is that each test takes approximately 20 minutes. Depending on the prevalence of HIV at the test site, the CD4 testing could become rate limiting.

In summary, the Pima CD4 finger stick test is an easy-to-use, portable, relatively fast device to test CD4+ T cell counts in the field. Issues of negatively-biased CD4 cell counts especially at higher absolute numbers will limit its utility for longitudinal immunologic response to ART. The high sensitivity and negative predictive value of the test makes it an attractive option for field use to identify patients eligible for ART, thus reducing delays in linkage to care and potentially ART initiation. Further studies to examine the impact of Pima on field HIV-tested patients and subsequent ART referral patterns is warranted.
